# Simulating Developmental and Individual Differences of Drawing Behavior in Children Using a Predictive Coding Model

**DOI:** 10.3389/fnbot.2022.856184

**Published:** 2022-06-20

**Authors:** Anja Philippsen, Sho Tsuji, Yukie Nagai

**Affiliations:** ^1^International Research Center for Neurointelligence (WPI-IRCN), The University of Tokyo, Tokyo, Japan; ^2^Institute for AI and Beyond, The University of Tokyo, Tokyo, Japan

**Keywords:** computational modeling, predictive coding, representational drawing, child development, recurrent neural network

## Abstract

Predictive coding has recently been proposed as a mechanistic approach to explain human perception and behavior based on the integration of perceptual stimuli (bottom-up information) and the predictions about the world based on previous experience (top-down information). However, the gap between the computational accounts of cognition and evidence of behavioral studies remains large. In this study, we used a computational model of drawing based on the mechanisms of predictive coding to systematically investigate the effects of the precision of top-down and bottom-up information when performing a drawing completion task. The results indicated that sufficient precision of both signals was required for the successful completion of the stimuli and that a reduced precision in either sensory or prediction (i.e., prior) information led to different types of atypical drawing behavior. We compared the drawings produced by our model to a dataset of drawings created by children aged between 2 and 8 years old who drew on incomplete drawings. This comparison revealed that a gradual increase in children's precision of top-down and bottom-up information as they develop effectively explains the observed change of drawing style from scribbling toward representational drawing. Furthermore, individual differences that are prevalent in children's drawings, might arise from different developmental pathways regarding the precision of these two signals. Based on these findings we propose a theory of how both general and individual development of drawing could be explained in a unified manner within the framework of predictive coding.

## 1. Introduction

An understanding of children's cognitive development and individual differences is pivotal in the cognitive sciences. However, considerable insights into the thinking processes of young children are still limited, because children often lack the linguistic and cognitive ability to explain their actions and decision-making processes. By observing and modeling children's natural behavior, it may be possible to propose hypotheses regarding how their behavior could be explained by underlying cognitive and perceptual mechanisms.

Drawing behavior is a widely used tool to gain insights into developmental processes (Thomas and Silk, 1990; Adi-Japha et al., 2010). It has been demonstrated that the drawings created by children reflect their developmental maturation (Thomas and Silk, 1990; Adi-Japha et al., 2010; Saito et al., 2014) and can aid in assessing how children perceive their environment (Chappell and Steitz, 1993; Barraza, 1999). Of particular interest is the emergence of representational drawings, which are drawings that depict objects or concepts (Saito et al., 2014). The development of representational drawing ability in children might reflect general cognitive maturation processes. However, the fundamental mechanism of its development, and the changes in perception and behavior that it involves are not well-understood.

The predictive coding idea has been proposed as a fundamental mechanistic account to explain how we integrate perceptual information with our prior experience in order to interpret the world around us and act in it (Rao and Ballard, 1999; Friston, 2009; Ciria et al., 2021). The theory suggests that the brain maintains an internal model of the world and constantly attempts to make predictions about what is occurring in the environment. If these top-down predictions differ from the actual bottom-up sensory sensations^1^, a prediction error arises.

This error, in turn, drives adaptation of the internal model, leading to learning or to the execution of actions that mitigate the prediction error (e.g., changing the perspective to resolve a visual illusion, or drawing the completion of a figure based on the expectations of the internal model). A key mechanism of this active inference or enactive predictive coding (Friston et al., 2010, 2011) is the integration of top-down and bottom-up information into posterior perception. It is assumed that the brain performs this integration using a Bayesian-optimal method, whereby how strongly prior information and current sensory information are taken into account depends on the precision of these two signals. Signals that are believed to be more precise would more strongly affect the resulting behavior, following the rules of Bayesian inference (Knill and Richards, 1996).

In recent years, computational studies have demonstrated that prediction could constitute a fundamental mechanism of cognition and an important driving factor during development (Nagai, 2019): Young children constantly face behavioral and perceptual challenges that require them to learn and update internal models of the environment (Cox et al., 2020). Experimental studies indicate that prediction errors, measurable as differential responses to more or less expected events (Kouider et al., 2015; Kayhan et al., 2019; Zhang et al., 2019), contribute to infants' learning from an early stage of development (Trainor, 2012; Ylinen et al., 2017). Additionally, the theory has also been applied to the study of individual differences, for example, to explain atypical perception and behavior in the context of developmental or psychiatric disorders (Gonzalez-Gadea et al., 2015; Sterzer et al., 2018; Lanillos et al., 2020; Angeletos Chrysaitis et al., 2021), or to provide a mechanistic explanation of individual differences of the precision of interoceptive perception (Ainley et al., 2016).

In this study, we used a computational model to address how the integration of top-down and bottom-up information might change as children develop by systematically modifying the two parameters expressing the precision of prior and current sensory information, respectively. Subsequently, we compared the drawings made by the model to drawings created by children in a drawing completion task where children could freely draw on partially drawn objects. Such a spontaneous drawing completion task was recently used to investigate the emergence of representational drawing ability in a systematic manner (Saito et al., 2014). Here, the drawing completion task is chosen because it can be considered as a prediction task: in order to decide what to draw, children use, on the one hand, the provided visual information which activates bottom-up processes. On the other hand, top-down processes are involved because previous experiences of the child or the model also influence drawing.

The computational model uses the ideas of predictive coding and is able to complete partial drawings based on the experience acquired during training. This model, first introduced in the study of Oliva et al. (2019), integrates the predictive learning of trajectories using a recurrent neural network with a module performing Bayesian inference to integrate the sensory input with the predictions of the network. This integration is performed flexibly depending on the precision of the sensory input and the network predictions. In our previous studies (Philippsen and Nagai, 2019, 2020b), we used this model to test how the strength of prior reliance affects drawing. The results indicated that adequate reliance on the prior was important for the successful completion of the stimuli. A weak reliance on prior information (if prior information was imprecise) was associated with scribbling behavior by the model. In contrast, an overly strong reliance on prior information (if prior information was highly precise) sometimes caused the model to misinterpret the presented pattern as a different pattern. Thus, imprecise prior information, causing a weak reliance on priors, might be the underlying reason why young children and non-human primates show scribbling behavior but do not create representational drawings as observed by Saito et al. (2014). One limitation of the previous study was that it took only the relative reliance on sensory and prior information into account: a stronger influence of prior information entailed a weaker influence on sensory information, and vice versa. However, not only the relative but also the absolute precision of the signals can be considered as crucial for explaining the resulting behavior. For example, it makes a difference if both signals are equally precise or equally imprecise. Another aspect that was not taken into account in the previous study is that the precision of sensory input, that was assumed to be fixed in our previous studies (Philippsen and Nagai, 2019, 2020b), is likely to change as a child develops. Specifically, various studies reported that sensory precision tends to increase with age (Sciutti et al., 2014; Karaminis et al., 2016). To account for the changes in sensory as well as in prior precision independently from each other, we extend the previous study by additionally modifying the reliance on the sensory input to obtain a more comprehensive understanding of the potential mechanism underlying the developmental pathway.

We compared the drawing of the model with drawings created by children aged between 2 and 8 years old that were recently obtained using a similar task design (Philippsen et al., 2020); children were presented with the drawing of an incomplete object such as a face or a house, and could freely draw on the presented stimulus. This comparison of data of the model to children's data at various ages enables us to postulate how children's precision might mature as they develop based on their displayed behavior.

Such a close comparison of psychological and computational data might be important to elucidate the underlying mechanisms of child development in the future. Additionally, we demonstrate the successful implementation of the computational model into the humanoid robot iCub and briefly discuss the system's potential to conduct human-robot interaction experiments for assessing human neurodiversity in future studies.

## 2. Background

An important mechanism of predictive coding is the integration of prior (top-down) and sensory (bottom-up) information. Human perception is considered to be almost Bayesian-optimal, relying on prior and sensory information based on the precision of these signals: more precise signals would more strongly influence the result. Consistent with this idea, previous studies have suggested that individual diversity might be caused by differences in the precision that individuals assign to sensory signals compared to their own predictions (Pellicano and Burr, 2012; Lawson et al., 2014; Lanillos et al., 2020). For instance, a stronger precision of the sensory signal compared to priors (hypo-prior) could account for the hypersensitivity in people with autism spectrum disorder (ASD) (Pellicano and Burr, 2012; Lawson et al., 2014), whereas a higher precision of priors (hyper-prior) could account for hallucinations in those with schizophrenia (Lanillos et al., 2020).

In additional, during development, children might integrate sensory information and priors in ways that differ from adult perception. While computational studies on developmental changes in precision are rare, behavioral experiments have been conducted to clarify how children's reliance on prior and sensory information may develop. However, the findings are to date not conclusive. In particular, younger children are often considered to possess weaker priors due to a lack of experience (Thomas et al., 2010; Stone, 2011). In contrast, Sciutti et al. (2014) found that children aged 7 years and older showed the same relative reliance on sensory and prior information as adults, but the absolute precision of these two signals was lower at a younger age. Some studies even report a slightly stronger reliance on priors in children. For example, Karaminis et al. (2016) reported that children aged 6–7 years still exhibited a stronger relative reliance on the prior to compensate for the imprecise sensory modality. As an alternative theory, it is also possible that children do not act in a Bayesian-optimal manner at all (i.e., they might randomly attend more strongly to prior or sensory information, regardless of the precision of the signals). However, the remarkable ability of children to perform statistical learning indicates that children already utilize an efficient sensorimotor integration and evidence accumulation mechanism (Saffran et al., 1996; Kirkham et al., 2002; Cox et al., 2020). As discussed in the studies of Sciutti et al. (2014) and Karaminis et al. (2016), the ability to rely on sensory and prior information according to their respective precision (i.e., being able to ignore imprecise signals) is a key mechanism that ensures robustness against environmental noise, and would, therefore, be important from an early age.

Our study assumes that Bayesian-optimal integration of prior and sensory information occurs at all ages, but that the precision attributed to the two signals may vary across development stages, in line with the view of Sciutti et al. (2014) and Karaminis et al. (2016). In particular, we systematically investigate how the precision of prior and sensory information, respectively, may affect behavior in the task of drawing completion. By comparing the simulated drawings to drawings created by children, we suggest a unified framework regarding how the precision of prior and sensory signals may account for the developmental and individual differences observed in children.

## 3. Methodology

This section introduces the computational framework (Section 3.1), motivates and describes the experimental design (Section 3.2) and describes the children's drawing dataset (Section 3.3).

### 3.1. Computational Model of Drawing Completion

**Figure 1:**
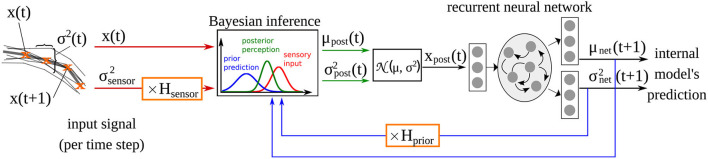
Computational model of drawing adopted from Philippsen and Nagai (2020b), and extended with the additional parameter *H*_sensor_ which scales the sensory precision $\sigma_{sensor}^{2}$ in the same way that *H*_prior_ scales the prior precision estimated of the network $\sigma_{net}^{2}$.

The computational model used here is adopted from our previous studies (Philippsen and Nagai, 2019, 2020b), in which we investigated how changes in the reliance on prior prediction can affect drawing ability. The model implements the idea of predictive coding by extending a recurrent neural network with a Bayesian inference module that integrates the sensory signal with the predictions of the network model at the input level (Oliva et al., 2019) as shown in Figure 1. The recurrent neural network acts as the internal model that generates predictions about the environment. Given input *x*^*t*^, it predicts the mean μ_net_ and the variance $\sigma_{net}^{2}$ of the sensory perception of the next time step. Simulated learning and inference is based upon a generative model comprising a likelihood and a prior. Based upon Gaussian assumptions about the prediction errors, the (log) likelihood is a function of the posterior mean and variance as well as the observed input **x**:


(1) $$\begin{array}{r} -\ln\;\left(L\right)=\overset{T-1}{\underset{t=0}{\sum }}\overset{D-1}{\underset{i=0}{\sum }}\left(\ln\left(2\pi {\sigma_{\text{net}}^{2}}^{t,i}\right)+\frac{{\left(x^{t+1,i}-{\mu_{\text{net}}}^{t,i}\right)}^{2}}{2{\sigma_{\text{net}}^{2}}^{t,i}}\right). \end{array}$$

Here, *T* is the total number of time steps, and *D* is the dimensionality of the input vector.

To enable the network to differentiate between different drawing stimuli classes (e.g., a face and a house), each stimuli independently uses a different set of initial neuron activations. Formally, the initial state ${\vec{u}}_{\left(s\right)}^{0}$ of stimuli class *s* is defined by the initial activations of the *N* neurons of the network's context layer at time step *t* = 0. The optimization criterion for enforcing this prior on the distribution of the initial states can be accordingly defined as


(2) $$-\ln(L_{\text{init}})=\sum_{s=0}^{S-1}\sum_{n=0}^{N-1}\left(\ln(2\pi v_{\text{dist}})+\frac{{\left(u_{\left(s\right)}^{0,n}-{\hat{\vec{u}}}^{n}\right)}^{2}}{2v_{\text{dist}}}\right),$$

where ${\hat{\vec{u}}}^{n}$ is the (learnable) mean of all initial states and *v*_dist_ (set here to *v*_dist_ = 10) is the predefined variance of the initial states. The network weights and the initial states are optimized during network training by maximizing *L*+*L*_init_ as proposed by Murata et al. (2013). This optimization process continues for a maximum of 30, 000 epochs (training is stopped earlier if no improvement is measured within the previous 5, 000 epochs).

After training, the output of the network's internal model (i.e., the posterior estimate μ_net_) constitutes the network's prior belief about the next time step. The prediction of the internal model μ_net_ is integrated with the sensory perception **x** in each time step according to the variance of the prediction $\sigma_{net}^{2}$ and the variance associated with the sensory input $\sigma_{sensor}^{2}$. The precision of the sensory signal $\sigma_{sensor}^{2}$ is fixed at a constant value of ~0.05, computed according to the actual variance present in the input signal (see Philippsen and Nagai, 2020b for details). The factors *H*_prior_ and *H*_sensor_ modify the variance terms when they differ from 1. *H*_prior_ was introduced in Philippsen and Nagai (2020b), where we modified this parameter, that expresses the variance (inverse precision) of the prediction. As a result, the model's behavior was modified so that it relied more weakly on its prior (in the case of larger variance, i.e., lower precision) or more strongly (in the case of smaller variance, i.e., higher precision), compared to a normal Bayesian-optimal manner of integration. In this study, we additionally modified *H*_sensor_, that implements aberrant sensory precision. The integration of sensory input with predictions follows the rules of Bayesian inference.


(3) $$\begin{array}{c} \sigma_{\text{posterior}}^{2}=\frac{\left(H_{\text{sensor}}\cdot \sigma_{\text{sensor}}^{2})\cdot (H_{\text{prior}}\cdot \sigma_{\text{net}}^{2}\right)}{\left(H_{\text{sensor}}\cdot \sigma_{\text{sensor}}^{2})+(H_{\text{prior}}\cdot \sigma_{\text{net}}^{2}\right)}, \end{array}$$

(4) $$\begin{array}{c} \mu_{\text{posterior}}=\sigma_{\text{posterior}}^{2}\cdot (\frac{\mu_{\text{net}}}{\left(H_{\text{prior}}\cdot \sigma_{\text{net}}^{2}\right)}+\frac{x}{\left(H_{\text{sensor}}\cdot \sigma_{\text{sensor}}^{2}\right)}). \end{array}$$

Using this mechanism, the input used for training the internal model is more strongly influenced by the input when the sensory modality is considered to be more precise than the prediction. If sensory input is less precise, the network automatically switches to a stronger focus on its own prediction.

### 3.2. Experimental Procedure

**Figure 2:**
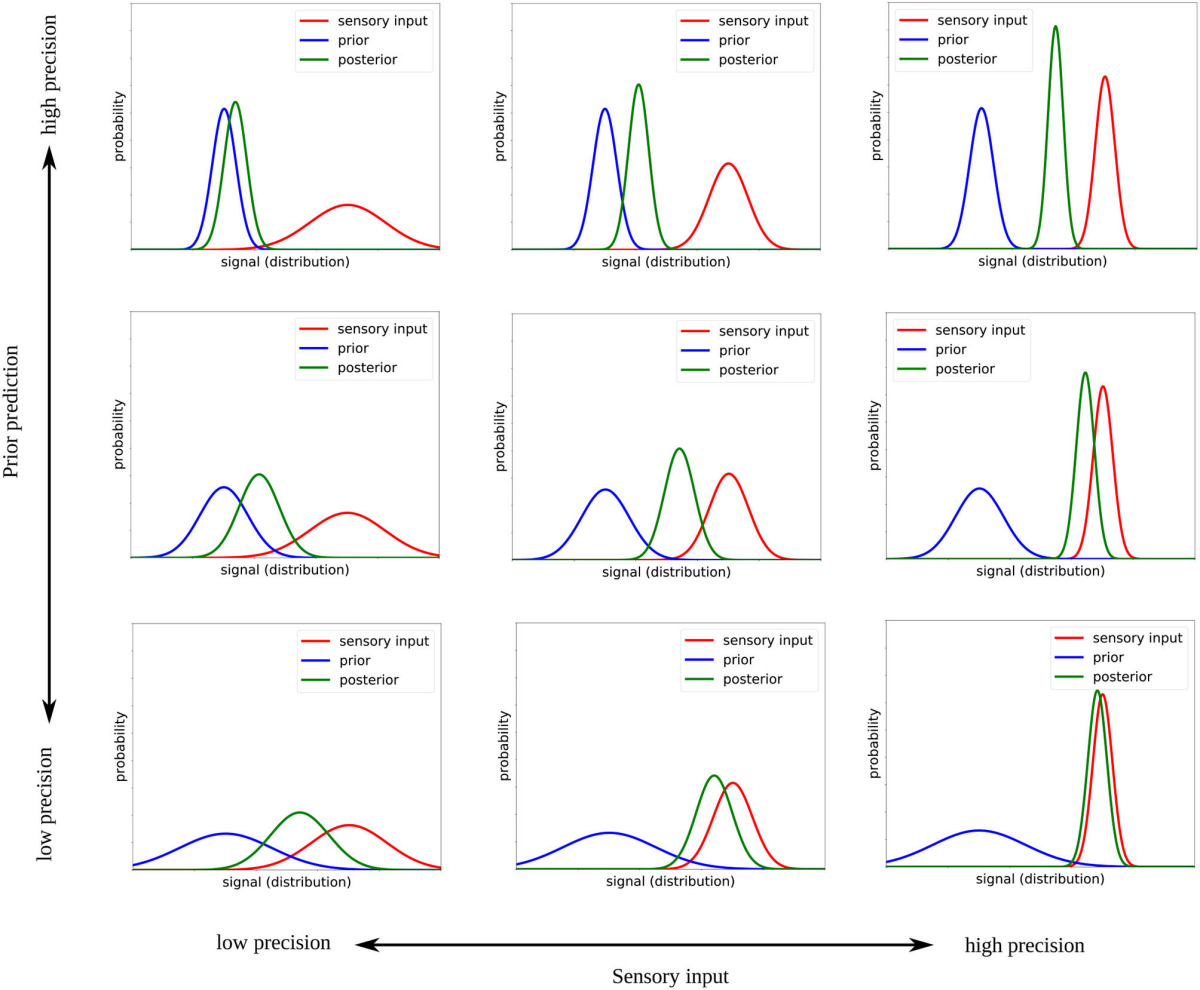
Illustration of how the Bayesian inference changes when the precision of the sensory input and the prior are modified.

The two-dimensional space explored in this study is shown in Figure 2 with exemplary designed prior and sensory precision values. The precision (i.e., inverse variance) of the sensor and prior distributions are gradually modified from low precision (bottom left corner) to high precision (top right corner). Note that at the diagonal from bottom left to top right, where the relative precision of both signals is the same, the mean of the posterior remains the same, however, the precision of that mean changes.

We modify the prior precision by a factor of *H*_prior_∈{0.001, 1, 1, 000} and the sensory precision by a factor of *H*_sensor_∈{0.001, 1, 1, 000}, where 1 means that the precision of the distributions corresponds to the network's estimated precision of its prior prediction or to the actual precision of the sensory input, respectively. A factor of 0.001 means that the distribution is thought to be much *more* precise (reduced variance), causing the model to pay more attention to this signal; a factor of 1, 000 means that the distribution is considered to be much *less* precise, thereby causing the network to pay less attention to this signal. These values correspond to the extreme values that were tested in Philippsen and Nagai (2020b) and were chosen in this study because they can best reveal the differences between the varying conditions.

**Figure 3:**
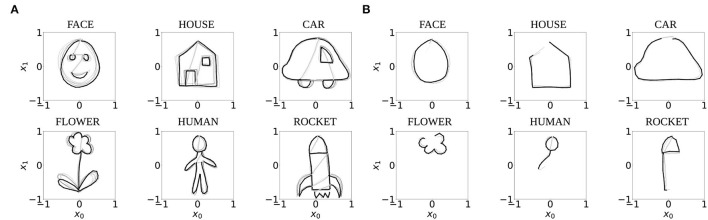
The seven trajectories used for training **(A)**, and the first 30 time steps of the three testing trajectories used to test the completion ability of the networks **(B)**. Black and gray lines indicate pen-down and pen-up lines, respectively. One example trajectory each is highlighted with bold lines for visual clarity.

The experiments in this study were performed with ten independently trained networks which were trained in our previous study (Philippsen and Nagai, 2020b) with normal prior and sensory reliance *H*_prior_ = *H*_sensor_ = 1 to perform one-step-ahead prediction of two-dimensional trajectories that represent six different drawing shapes: FACE, HOUSE, CAR, FLOWER, HUMAN, and ROCKET. To create this data set, ten trajectories of each shape were drawn manually by a human subject and downscaled to consist of 90 time steps each. Seventy percent of these trajectories were used for training the network (Figure 3A), and 30% for evaluating the model's completion ability. Of these testing trajectories, only the first third of the trajectories (30 time steps) were presented to the network as shown in Figure 3B.

During recognition and generation, the altered parameter values *H*_prior_ and *H*_sensor_ were used to test how this affects the network's ability to complete the drawings. The completion is performed in two steps. First, the network tries to recognize the incomplete trajectory consisting of the first 30 time steps by inferring which initial configuration of neuron activations would best account for the observation of the presented trajectory (recognition step). For this purpose, the backpropagation-through-time algorithm was applied for 100 epochs to optimize with regard to the initial condition while maintaining the network weights fixed (Murata et al., 2014). The inferred initial configuration is then used as a starting point for generating the full trajectory (generation step). This way of completing the trajectories is inspired by the principle of predictive coding and is suggested to be analogous to how humans would infer the underlying causes of an observation while acting in a way that minimizes the prediction error (Murata et al., 2014).

Further details about the implementation can be found in Philippsen and Nagai (2020b). The source code for this experiment is provided as part of the GitHub repository of Philippsen and Nagai (2020b)^2^.

### 3.3. Child Drawing Data Set

An important aim of this study is to use computational data to make predictions about the underlying cognitive mechanisms that might drive the behavior of children over the course of their development. For this purpose, it is not sufficient to look at synthetic data; instead, comparisons to behavioral data of children are required to connect computational findings to actual behaviors.

**Figure 4:**
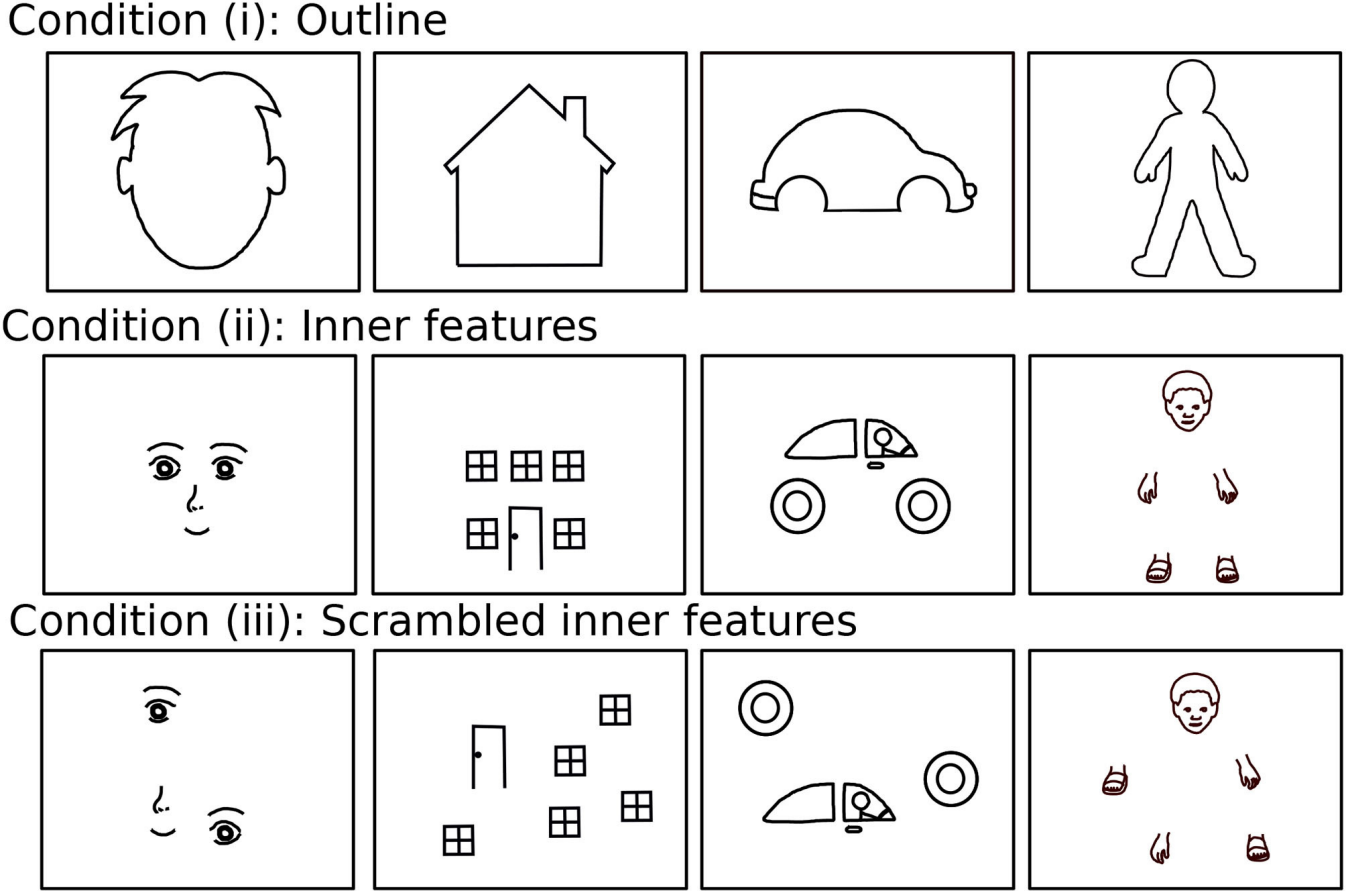
The 12 stimuli of which six were presented to each of the children, consisting of four different stimuli categories (face, house, car, and human figure) and three presentation conditions (outline, inner features, and scrambled inner features).

Here, we use a drawing data set of drawing completion that was recorded recently using a similar task design as that used in the computational study (Philippsen et al., 2020). Children were presented with incomplete stimuli, representing animate, or inanimate objects such as a face, a house, or a car where crucial parts of the object were missing, such as facial features or windows (see Figure 4 for the full set of stimuli). Note that the stimuli for the child experiment provided more details than the stimuli of the computational study (e.g., the outline of the ears and the hair is shown for the face stimulus) to make them child-friendly and to allow the children to better recognize the intended shape. Furthermore, stimuli were presented in the child study in three different conditions where the outline, inner features, or scrambled inner features were shown, respectively. The condition with scrambled inner features was added for investigating the relation of individual differences to autistic traits (Philippsen et al., 2020, under review). In the present study, however, we do not consider differences between the conditions and the neural network is only trained on stimuli of the outline condition as shown in Figure 3A.

Data from 104 children (62 males and 42 females) aged between 2 and 8 years old (average age 4 years and 9 months) were collected in a science museum, resulting in a total of 621 drawings. Drawings were performed on a tablet PC using blue color to distinguish the child-drawn parts from the presented parts. Children were instructed to draw whatever they liked, and each child could draw up to six different stimuli.

**Figure 5:**
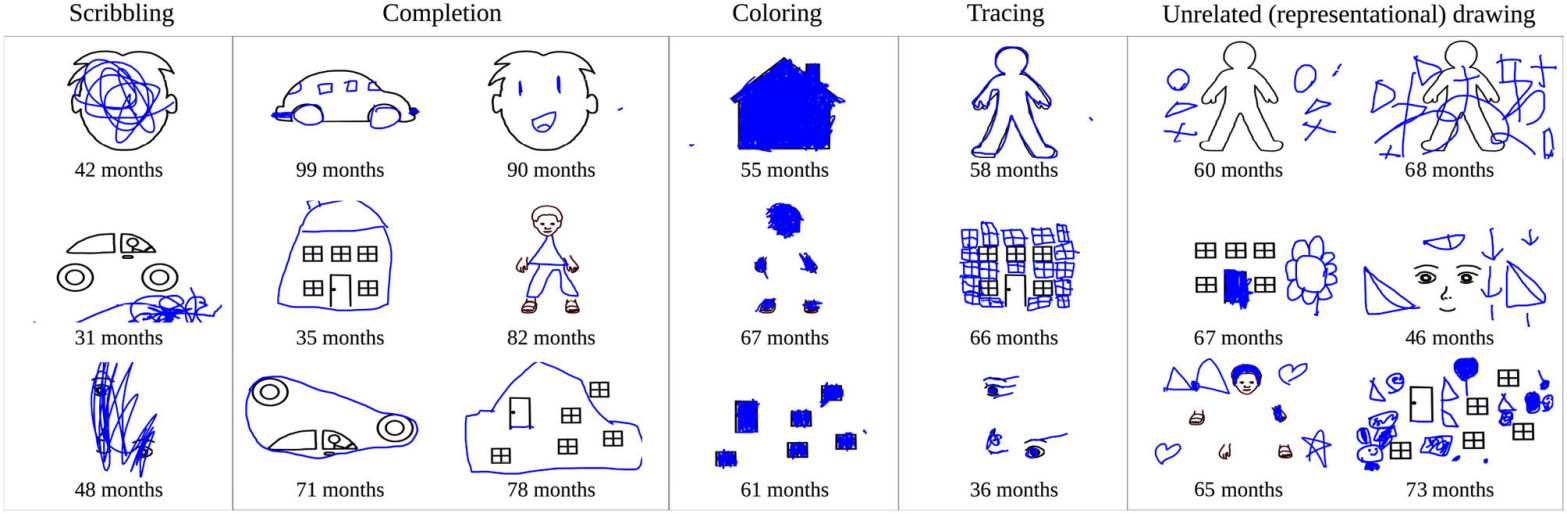
Example drawings of children that were categorized as scribbling, completion, coloring, or tracing by adult raters, and examples of drawings that seem unrelated to the presented stimuli but instead show objects or simple shapes.

**Figure 6:**
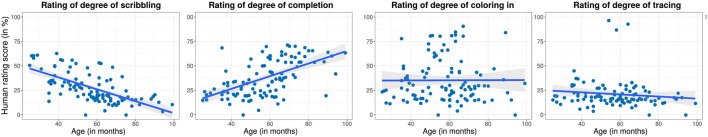
Overview of human ratings of the degree to which children showed scribbling, coloring in, tracing, and completion in their drawings, between 0% (not at all) and 100% (strongly) at different ages. Every point represents the mean score for a single child, the line shows the linear regression.

A previous analysis of this data set revealed that children show large developmental and individual differences in their responses to this task. Specifically, ratings of human adults were collected to assess the overall trend of children's development (Philippsen et al., 2020). Younger children tended to show a higher degree of scribbling and a lower degree of completion of the stimuli, whereas the reverse trend was observed in older children. Children also displayed other drawing styles such as the coloring in of shapes, tracing, or copying presented parts of the drawing, or even the drawing of objects that did not show any obvious relationship to the presented stimulus. Figure 5 shows examples of children's drawings that are representative of these different drawing styles. Figure 6 provides an overview of the human rating assessment of children's drawing behavior that was conducted in Philippsen et al. (2020), plotted against the children's age. Children showed a decrease in scribbling and an increase in completion with increasing age, whereas coloring and tracing behavior could be observed at all ages.

## 4. Results: How Reliance on Prior and Sensory Information Affect Drawing

This section presents the results of the computational study. First, we explain the results of the simulation qualitatively for one of the trained networks, and then confirm the observed trends by averaging across all ten trained networks. Second, we briefly present the implementation of the drawing experiment into a physical robot and discuss potential future studies for investigating human neurodiversity.

### 4.1. Simulation Results

**Figure 7:**
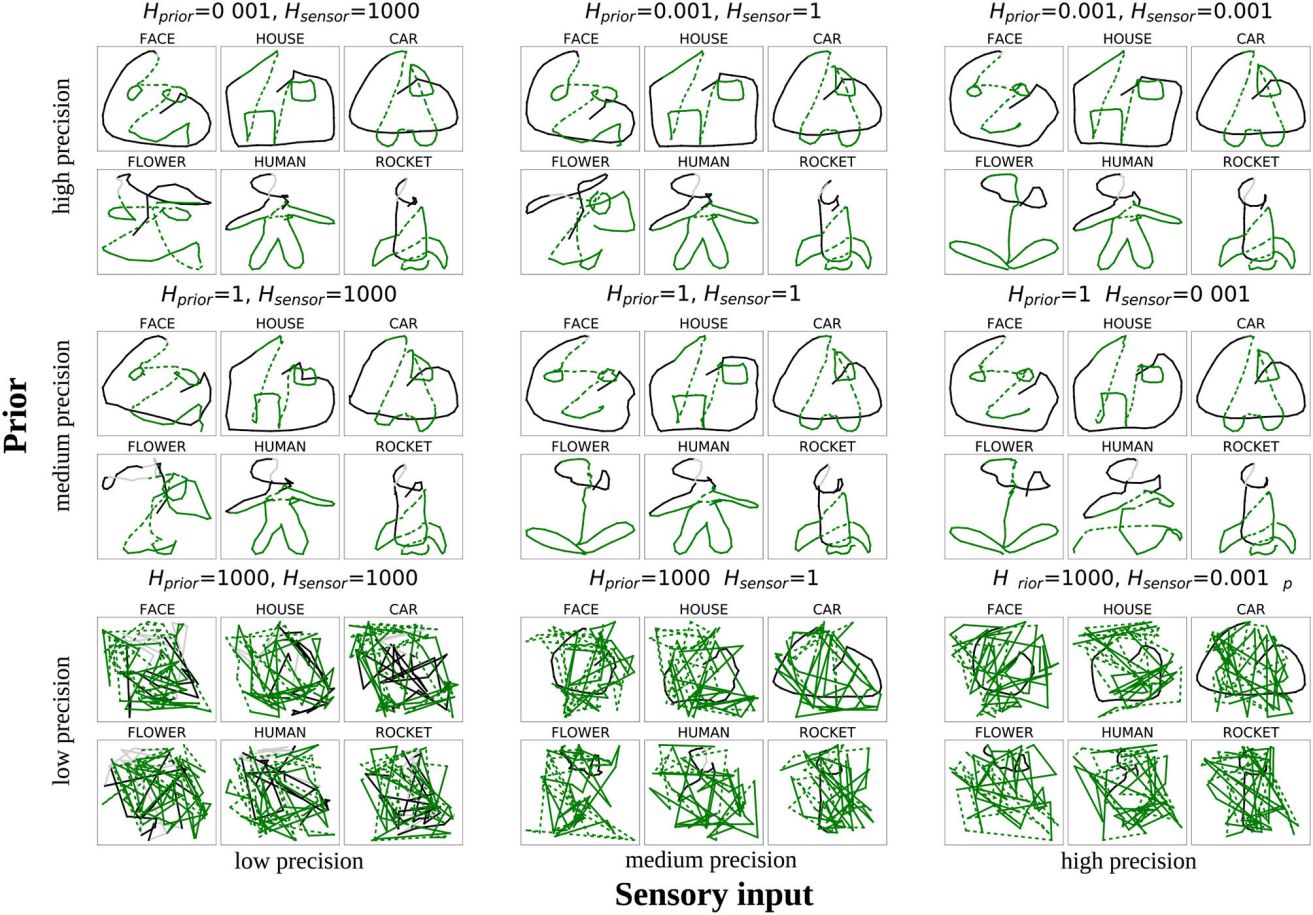
Drawings produced by one network when modifying the variance of sensory input *H*_sensor_ (x-axis) and the variance of prior predictions *H*_prior_ (y-axis) by a factor of 1, 000 (low precision), 1 (normal precision), or 0.001 (high precision). The black and gray lines indicate how the network could reproduce the presented part (cf. Figure 3B), the green lines show the network's completion ability (solid lines for pen-down, dashed lines for pen-up drawing).

In Figure 7, an example is shown how one of the networks performed for the nine different constellations of *H*_prior_ and *H*_sensor_. The black lines show the first 30% of the trajectories, indicating whether the model is able to trace the presented part of the stimulus. The green lines show how the model completed the stimulus.

It can be observed that the drawings produced with normal reliance on both signals (middle of Figure 7) were correctly completed according to the training data. The same was true if the precision of both signals was increased (top right corner). In contrast, when there was a drastic reduction in the precision of both signals (bottom left corner), the model did not show any meaningful behavior; it could neither follow the presented lines nor complete the pattern, but rather instead, it produces random movements.

The top-left and bottom-right corners show cases where the network only considered one of the two integrated signals to be precise, whereas the other one was considered to be imprecise. In the bottom-right corner of Figure 7 we can see the result of the model simulations when an overly strong reliance was placed on the sensory signal, and little attention was directed to the prior signal. Initially, the performance looks very similar to the performance in the bottom-left corner. The difference between these two conditions lies with the black lines corresponding to the behavior of the model while observing the presentation of the first 30 time steps. Whereas the network showed only scribbling behavior in the bottom-left corner, in the bottom-right corner, the network is able to trace the presented (black) lines. This result demonstrates that a sufficient degree of precision of the prior information is required to allow the model to complete a partial drawing, but because of the precise bottom-up signal, the model can perform more low-level behaviors such as following a presented trajectory.

The opposite case is displayed at the top left corner of Figure 7, in which sensory information is considered to be imprecise, but prior information is precise. In this case, the model can follow existing lines, although it is less accurate than with higher sensory precision. Owing to sufficiently precise prior information, the model can also complete the shapes in many cases. However, because of the relatively low reliance on sensory signals, the completed shape did not always accurately fit the intended pattern. Specifically, in the example shown, the model drew a face pattern instead of a flower to complete the bottom-left shape. Such a drawing of a shape that does not fit the intended shape is caused by an excessively strong reliance on the priors combined with insufficient reliance on the sensory input, which leads to an over-weighting of the network's confidence in its own prediction. Owing to the low precision of the sensory input, this prediction is not always correct, leading to confusion between shapes.

In summary, two main findings can be extracted from this analysis: First, the model creates random trajectories when insufficient prior information is available—a behavior that resembles scribbling. Second, the low precision of sensory input together with precise priors seems to cause the network to confuse the shapes with each other in some cases.

To measure whether these tendencies hold for the entire dataset, we evaluated the drawings that were produced by all 10 networks. Each network completed the six patterns three times, using the test dataset (cf. Figure 3B).

**Figure 8:**
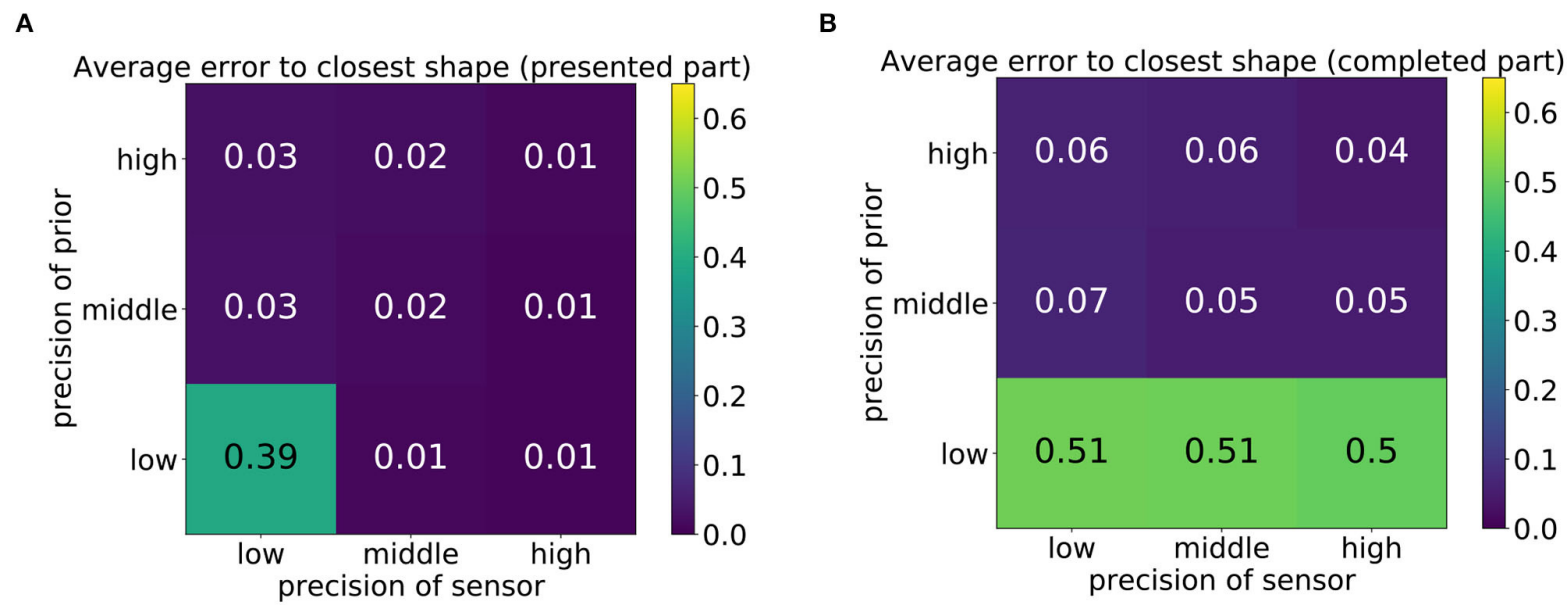
Average mean square error of the network's drawn shape to the shape that best matches the shape when comparing either the first 30 time steps (presented part) **(A)** or the last 60 time steps (completed part) **(B)**.

First, the distances (mean square error) of the produced trajectories to all training trajectories were computed to measure the overall quality of the produced drawing. The average error of the produced drawings to the closest training trajectories is displayed in Figure 8, where Figure 8A shows the error computed for the first third of the trajectory and Figure 8B shows the error computed on the last two thirds, that is, the network's completion, which was performed without visual guidance. The nine tiles of the matrix correspond to the nine parameter constellations shown in Figure 7.

It can be observed that the error was small in most cases but high in the bottom-left corner of Figure 8A and in the entire bottom row of Figure 8B. This high error was caused by the scribbling-like behavior observed in Figure 7. Whereas with sufficient precision of the sensory input, scribbling only occurred in the completed part of the trajectory (Figure 8B), a low precision of both signals caused the model to scribble regardless of the presence of sensory input.

**Figure 9:**
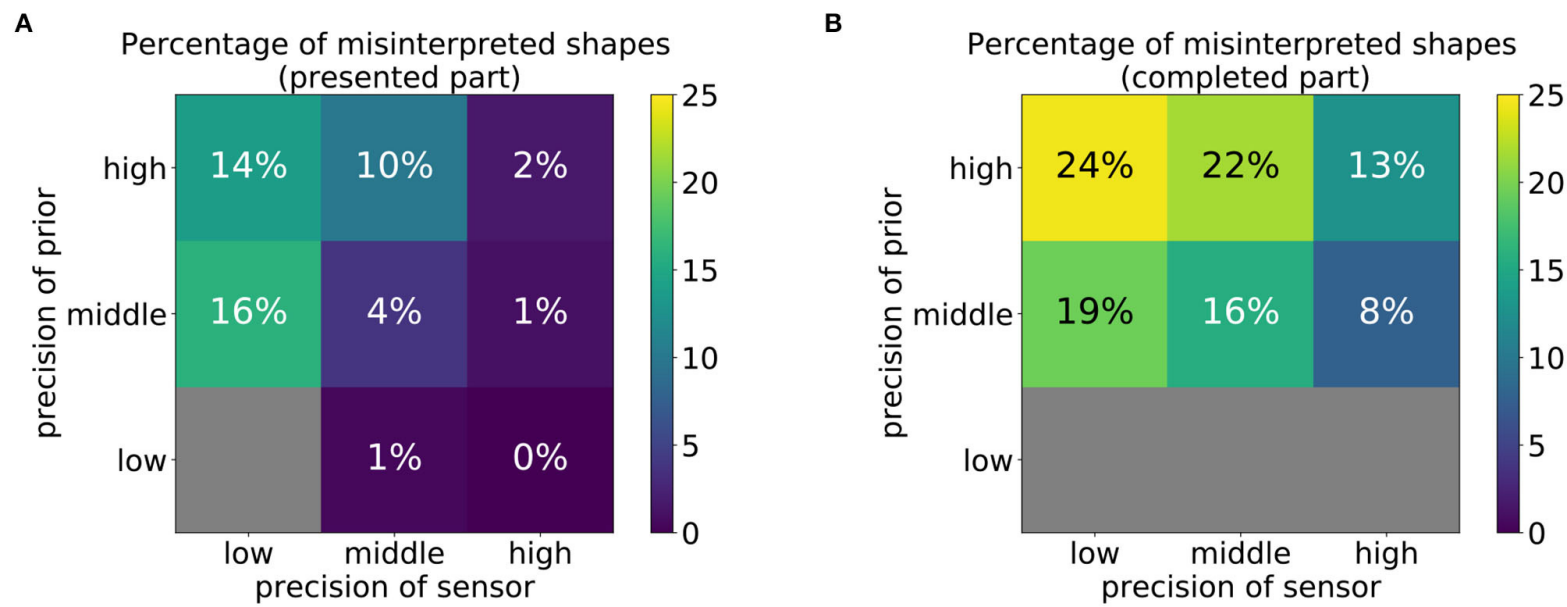
Percentage of shapes that were misinterpreted as another pattern that was not the intended pattern, computed using **(A)** the first 30 time steps (presented part) or **(B)** the last 60 time steps (completed part). Gray color indicates that this constellation led to random scribbling (cf. Figures 7, 8) such that the percentage cannot be reliably computed.

Next, we determined for each parameter constellation, how often the model misinterpreted the presented shape as a different shape. Specifically, we counted how often the training shape closest to the produced drawing corresponded to a shape that was *not* the intended shape. The percentages of such misinterpretations are shown in Figure 9. Note that the tiles that displayed an extremely high value in Figure 8 were left out in this analysis because an error of 0.3 or higher was found, indicating that the drawn shape was not similar to any of the training shapes. In these cases, the model failed to perform any type of representational drawing; therefore, no misinterpretations can occur.

The results in Figure 9 show that misinterpretations occur at a higher percentage at the top-left corner of both matrices (Figures 9A,B). This confirms that they are more likely to occur if the relative reliance on prior information is stronger than that on sensory information. Conversely, if the reliance on sensory information is more precise than that on prior information, the rate of misinterpretations even declines compared to the balanced conditions. However, the performed strokes generally tend to be less smooth (cf. middle right figure of Figure 7).

### 4.2. Physical Robot Implementation

Additionally to the simulation, the drawing study was also implemented in the physical humanoid robot iCub to enable a human to interact with the robot while observing the differences in drawing behavior when the precision of the prior changes.

**Figure 10:**
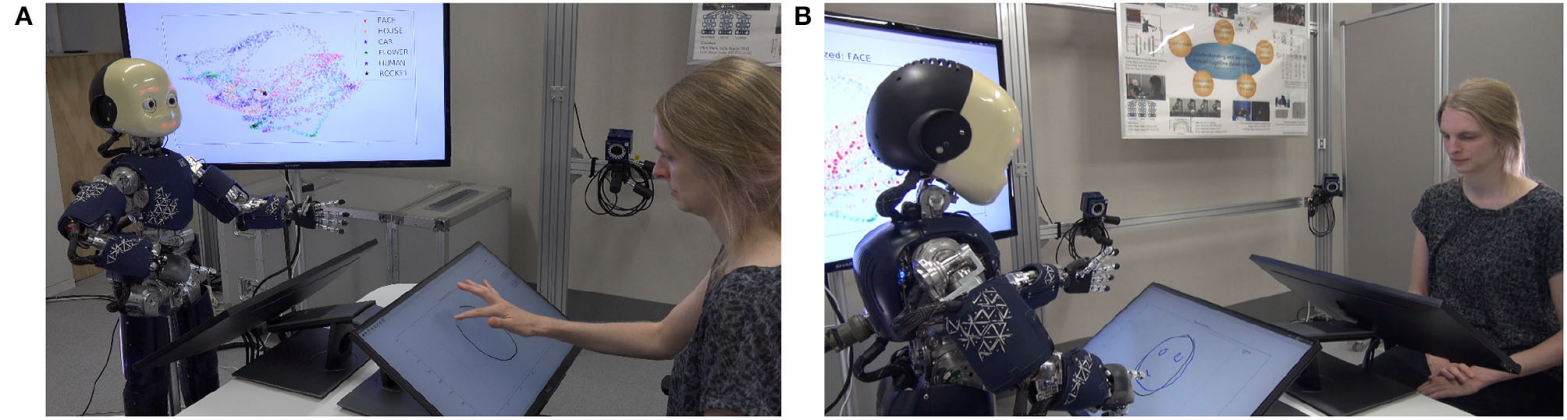
The experimental setup of the demonstration of the system implemented on the iCub robot. **(A)** The human starts to draw a line and **(B)** the robot completes the drawing.

As shown in Figure 10, the human and the robot sit face-to-face to each other; both have their own touch screen in front of them on which the drawing of both agents is displayed. The human starts the experiment by drawing the beginning of one of the shapes that were learned by the network. The robot, then, completes the shape. Internally, the trained neural network is used for generating the completed trajectory, as in the simulation experiments, and the iCub's arm movements are calculated accordingly and synchronized with the line that gradually appears on the screen. To reduce the implementation effort, the robot is not actually touching the screen, however, by accordingly synchronizing the movement, the observer is provided with the impression that the robot is drawing the line by itself. In addition, on a separate screen the internal representation of the neurons of the network are displayed live during the robot's drawing (see the screen behind iCub in Figure 10A). As explained in detail in Philippsen and Nagai (2020b), this figure visualizes the “cognitive” mechanism that the robot applies when solving the completion task.

In its current form, the system mainly serves demonstration purposes, namely, to make the effect of the change of the precision of the robot's prior easily understandable for the human observer. However, in the future, the system could be used for conducting human–robot interaction experiments of collaborative drawing with the purpose to investigate the cognitive mechanisms of the human. This can be achieved by switching the role of the human and the robot such that the human is requested to complete the drawing of the robot. While the currently used drawing shapes might be too complex for the purpose of a systematic investigation, as the diversity of child drawings on the stimuli indicates (Philippsen et al., 2020), simpler drawing shapes might be applicable for this purpose. For instance, a simple drawing task has been used in Murata et al. (2019) to visualize cognitive characteristics of a large group of people. Their study found correlations of drawing behavior to psychiatric symptoms. Our setup might be particularly useful for assessing developmental changes and human neurodiversity with respect to social interactions due to the additional factor of physical embodiment of the system. For instance, the system could be used to extend the iCub experiment of Mazzola et al. (2020) where the prior of human perception (in form of the central tendency effect) was measured in individuals interacting with the iCub in a social situation.

## 5. Interpretation of Results and Connection to Child Data

In the presented analysis, we explored how gradual changes in the sensory and prior precision affected the drawing behavior of the computational model. We found that the completion ability increased with higher precision of the sensory and prior signal. The high precision of both signals is associated with successful completion of representational drawings, whereas too low precision leads to random trajectories that resemble the scribbling behavior that young children sometimes display.

When we compare these findings to children's drawings, it seems that a gradual increase in the precision of sensory and prior information over development could account for the developmental change in children's drawing behavior, ranging from scribbling-like behavior at a young age to accurate completion behavior later on. However, children's behavior was not limited to these two drawing styles. Instead, the drawings were more diverse, including drawing styles such as tracing, coloring, or drawing of seemingly unrelated patterns. As shown in Figure 6, these drawing styles did not show any clear correlation with the child's age. Following the findings from the computational model, some of these drawing styles might be explained by an imbalance of sensory and prior information. Specifically, the two extreme cases where either sensory or prior precision is high and the other signal's precision is low reveals typical behavior patterns that could have their equivalent in the children's drawing data. With high sensory precision but insufficient prior precision, the model showed scribbling behavior but was able to trace the presented part of the trajectory. Children who traced the presented stimuli, but did not show any representational drawing, thus, could have overly relied on the bottom-up signal while not making use of prior information. In the opposite case, with low sensory but high prior precision, the model was able to complete drawings but sometimes drew shapes which did not correspond to the presented shape. Similarly, children sometimes drew objects or shapes on top or next to the presented stimulus that did not have an obvious connection with the presented shape. One possible explanation for this tendency is that children might have relied more strongly on their priors and neglected sensory information in some cases.

**Figure 11:**
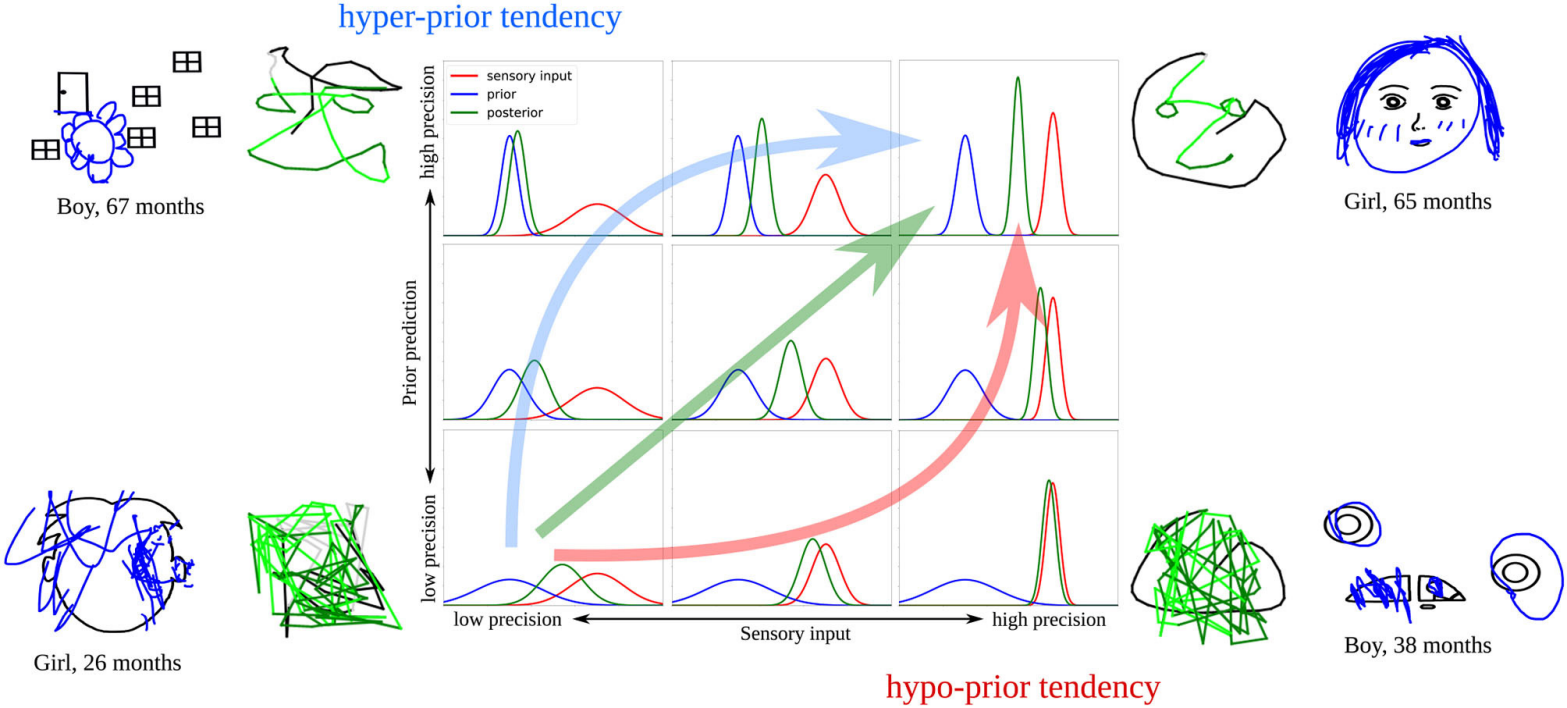
Illustration of the proposed developmental pathways that might account for the drawing behaviors observed in the child study. The green arrow shows a parallel increase of sensory and prior precision, the red arrow illustrates how precision would increase with a hypo-prior tendency, the blue arrow with a hyper-prior tendency. Examples of model results and child drawings representative for these cases are displayed.

Figure 11 summarizes these ideas. Developmental changes observed in the child study (increase in completion and decrease in scribbling), together with the simulations of the computational model, suggest that there is a general trend across development leading from low to high precision in both signals. Such a parallel maturation of the precision of both modalities could, therefore, constitute the optimal developmental pathway (green arrow in Figure 11).

However, it is likely that the maturation of sensory and prior precision does not always occur in parallel, as in the optimal case. As a result, an imbalance might occur, causing the model to over- or underestimate the precision of either the prior or the sensory signal at certain points in the development. Previous studies revealed inconclusive results regarding the maturation of sensory and prior precision across various age groups (Thomas et al., 2010; Stone, 2011; Sciutti et al., 2014; Karaminis et al., 2016), which leads to the possible explanation that each child follows an individual pathway—an explanation that could account for the diversity that is observable in children's drawings.

Our simulations suggest that some of the displayed drawing styles could be the result of a divergence from the optimal developmental pathway, leading to a stronger reliance on prior information (hyper-prior tendency, blue arrow in Figure 11) or sensory information (hypo-prior tendency, red arrow in Figure 11). Specifically, children in Philippsen et al. (2020) might have applied different strategies when integrating prior and sensory information. Some children drew figures that seemed unrelated to the presented stimulus, relying more strongly on their own priors, while ignoring the presented sensory information. Other children might have relied more strongly on the presented bottom-up information, which in our simulations was associated with scribbling or tracing behavior.

In conclusion, our findings suggest that children's development leads them from low to high precision, and that not only the relative but also the absolute precision of both signals plays an important role and could be related to individual drawing differences that are observable in children's drawings.

## 6. Conclusion

In this study, we investigated how the precision of prior and sensory signals influences behavioral outcomes in a drawing completion task. A computational model based on the predictive coding theory was employed to systematically analyze how these two precision values may affect drawing. By comparing the model's behavioral output with drawings produced by children aged between 2 and 8 years in the context of a similar task, we propose a theory of how precision of prior and sensory signals may develop with increasing age in children.

Our findings indicate that a gradual increase in the precision of both signals could account for the decrease in scribbling and the increase in the completion of drawings in the child study. Moreover, different individual pathways in the development that might lead to a temporary overweighting of either the precision of the prior or of the sensory signal could account for some of the individual differences that were observed in children's drawings. Therefore, the predictive coding theory and, more precisely, changes in the precision of the prior and sensory signals, could account for the developmental and individual differences of children in the context of the presented task.

In the future, experimental studies are required that systematically analyze the precision that children attribute to prior and sensory information over their development to confirm this hypothesis. Longitudinal studies are particularly important in this regard to reveal whether individual children consistently follow one of the proposed developmental pathways in Figure 11, or instead show higher variability compared to adults, causing the presence of hypo- and hyper-prior tendencies in the same individual.

The novelty of this study is that it directly compared the data obtained from the simulation with children's drawing data. Both the model and the child experiment used the same underlying task design. However, whereas the child study was designed to replicate the completion task of the computational model as closely as possible, there are a number of differences between these studies that should be acknowledged.

The first difference lies in the way that the model perceives the world, compared to human perception. Specifically, for the model, the first part of the picture was revealed continuously as a trajectory, whereas it was presented to the children before any action. This difference is caused by the nature of the computational model that requires a recurrent neural network at its core in order to be able to generate time-dependent predictions and implement the ideas of predictive coding. This design should be improved in the future to allow for a more human-like perception.

A second limitation is that, in contrast to a child, the model is not creative and cannot produce any other meaningful output patterns than those for which it has been previously trained. As a result, only a subset of the drawing styles that children displayed, could be replicated by the network. For example, the model always completes drawings in a similar way whereas children could complete drawings in many different ways—a difference not accounted for in this study. Children also sometimes colored in the shapes, which is a behavior that cannot be easily simulated within the current setting: the model displays scribbling behavior, but it cannot be restricted to draw only inside a particular region of the drawing plane.

Also, it should be noted that our interpretation of the children's drawing data constitutes only one possible explanation. In particular, the task instructions that were provided to the children were not strict: the children were not explicitly asked to complete the drawings, but instead to draw whatever they liked. Thus, it is possible that children that did not complete the drawing, instead found other reasonable interpretations of the task and created the drawings according to this interpretation.

Finally, an important limitation is that the model cannot direct attention to specific regions of the drawing. Therefore, in the simulations, it was necessary to artificially differentiate two cases: drawing on the first part of the drawing that was presented to the model, and completing the second part without the availability of any input. This computational design was chosen to obtain a complete impression of the capabilities of the network. However, because of this design, the computational model always drew the full trajectory consisting of the presented part and the completed part, whereas most children completed the drawing without additionally tracing the existing lines, although they could do so if they chose. Such differences between the computational model and children's behavior limit the degree to which they could be compared and should be addressed in future studies. For example, the child experiment could be modified to fit the computational study more closely by letting the drawing appear gradually on the screen, which might motivate children to also display tracing behavior. It could also be considered to analyze the model data analogously to how the child data was analyzed in previous studies (Philippsen et al., 2020, under review) to further verify the interpretation of this simulation and eventually strengthen the link between the child and the model data.

Being based on the principle of predictive coding, these results could also have a potential impact on gaining an understanding of the mechanistic causes of neurodevelopmental disorders. Recently, several studies suggested that an imbalance in the integration of prior and sensory information might constitute a root cause for developmental and psychiatric disorders (Gonzalez-Gadea et al., 2015; Idei et al., 2018; Sterzer et al., 2018; Lanillos et al., 2020; Philippsen and Nagai, 2020a). However, experimental evidence is not consistent. In particular, a recent study with individuals with ASD did not find any correlation between the usage of prior information and autistic traits (Angeletos Chrysaitis et al., 2021). Our simulation results suggest that the interplay of using sensory and prior information might explain individual differences in behavior observed throughout development, but it is a subject of future study to assess whether our proposed method can elucidate systematic differences between individuals with and without ASD. Sterzer et al. (2018) suggested that the usage of prior information might depend on the sensory modality involved and the hierarchical level of processing, indicating that the specific task design is crucial and could account for inconsistent evidence. Our findings suggest that additionally developmental stages play an important role, and should be considered carefully in future research.

In conclusion, our study proposed a novel hypothesis on the developmental pathways of children based on the predictive coding theory. We demonstrated that the hypothesis is plausible using a computational model that reflects the behavioral data of children. Such a close connection between computational and behavioral studies may be a key component of future research as it opens up new approaches to study the underlying cognitive mechanisms involved in child development.

## Data Availability Statement

The datasets presented in this study can be found in online repositories. The name of the repository and accession number can be found at: GitHub, https://github.com/aphilippsen/drawingcompletion.

## Ethics Statement

The studies involving human participants were reviewed and approved by Research Ethics Committee of the Office for Life Science Research Ethics and Safety at The University of Tokyo. Written informed consent to participate in this study was provided by the participants' legal guardian/next of kin. Written informed consent was obtained from the individual(s) for the publication of any identifiable images or data included in this article.

## Author Contributions

AP, ST, and YN conceived the experimental design, analyzed, and interpreted the results. AP and YN designed the computational experiments. AP implemented and conducted the computational experiments and drafted the manuscript. YN and ST reviewed the manuscript. All authors read and approved the final manuscript.

## Funding

This work was supported by JST CREST “Cognitive Mirroring” (Grant Number: JPMJCR16E2), Japan, by JST CREST “Cognitive Feeling” (Grant Number: JPMJCR21P4), Japan, by Institute for AI and Beyond, The University of Tokyo, Japan, and by the World Premier International Research Center Initiative (WPI), MEXT, Japan.

## Conflict of Interest

The authors declare that the research was conducted in the absence of any commercial or financial relationships that could be construed as a potential conflict of interest.

## Publisher's Note

All claims expressed in this article are solely those of the authors and do not necessarily represent those of their affiliated organizations, or those of the publisher, the editors and the reviewers. Any product that may be evaluated in this article, or claim that may be made by its manufacturer, is not guaranteed or endorsed by the publisher.

## Footnotes

^1^There is an ongoing discussion about the roles of top-down and bottom-up signals and how they should be formalized (Rauss and Pourtois, 2013). Here, we adopt the view that top-down signals contain information that has been acquired beforehand, for example, through experience or *via* training (Barlow, 1997; Rauss and Pourtois, 2013).

^2^ https://github.com/aphilippsen/drawingcompletion
